# Detrended Fluctuation Analysis of Heart Rate Dynamics Is an Important Prognostic Factor in Patients with End-Stage Renal Disease Receiving Peritoneal Dialysis

**DOI:** 10.1371/journal.pone.0147282

**Published:** 2016-02-01

**Authors:** Jiun-Yang Chiang, Jenq-Wen Huang, Lian-Yu Lin, Chin-Hao Chang, Fang-Ying Chu, Yen-Hung Lin, Cho-Kai Wu, Jen-Kuang Lee, Juei-Jen Hwang, Jiunn-Lee Lin, Fu-Tien Chiang

**Affiliations:** 1 Division of Cardiology, Department of Internal Medicine, National Taiwan University Hospital Hsin-Chu Branch, Hsin-Chu, Taiwan; 2 Division of Nephrology, Department of Internal Medicine, National Taiwan University College of Medicine and Hospital, Taipei, Taiwan; 3 Division of Cardiology, Department of Internal Medicine, National Taiwan University College of Medicine and Hospital, Taipei, Taiwan; 4 Department of Medical Research, National Taiwan University Hospital, Taipei, Taiwan; 5 Institute of Epidemiology and Preventive Medicine, College of Public Health, National Taiwan University, Taipei, Taiwan; 6 Department of Laboratory Medicine, National Taiwan University Hospital, Taipei, Taiwan; Hospital Universitario de La Princesa, SPAIN

## Abstract

**Background and Objectives:**

Patients with severe kidney function impairment often have autonomic dysfunction, which could be evaluated noninvasively by heart rate variability (HRV) analysis. Nonlinear HRV parameters such as detrended fluctuation analysis (DFA) has been demonstrated to be an important outcome predictor in patients with cardiovascular diseases. Whether cardiac autonomic dysfunction measured by DFA is also a useful prognostic factor in patients with end-stage renal disease (ESRD) receiving peritoneal dialysis (PD) remains unclear. The purpose of the present study was designed to test the hypothesis.

**Materials and Methods:**

Patients with ESRD receiving PD were included for the study. Twenty-four hour Holter monitor was obtained from each patient together with other important traditional prognostic makers such as underlying diseases, left ventricular ejection fraction (LVEF) and serum biochemistry profiles. Short-term (DFAα1) and long-term (DFAα2) DFA as well as other linear HRV parameters were calculated.

**Results:**

A total of 132 patients (62 men, 72 women) with a mean age of 53.7±12.5 years were recruited from July 2007 to March 2009. During a median follow-up period of around 34 months, eight cardiac and six non-cardiac deaths were observed. Competing risk analysis demonstrated that decreased DFAα1 was a strong prognostic predictor for increased cardiac and total mortality. ROC analysis showed that the AUC of DFAα1 (<0.95) to predict mortality was 0.761 (95% confidence interval (CI). = 0.617–0.905). DFAα1≧ 0.95 was associated with lower cardiac mortality (Hazard ratio (HR) 0.062, 95% CI = 0.007–0.571, P = 0.014) and total mortality (HR = 0.109, 95% CI = 0.033–0.362, P = 0.0003).

**Conclusion:**

Cardiac autonomic dysfunction evaluated by DFAα1 is an independent predictor for cardiac and total mortality in patients with ESRD receiving PD.

## Introduction

High cardiovascular (CV) morbidity and mortality are well documented in patients with chronic kidney disease (CKD) and end stage renal disease (ESRD) receiving dialysis.[1] Sympathetic over-excitation plays an important role in the pathogenesis leading to the development of cardiovascular complications.[2] In recent years, heart rate variability (HRV) parameters derived from the beat-to-beat heart rate dynamics have been used as markers of autonomic modulation. ^3^For patients with CKD/ESRD, several HRV parameters based on linear analysis such as Fourier transform had been verified to predict patient outcomes. ^4, 5^ For example, decreased HRV measured by 24-hour ambulatory ECG is an independent predictor of mortality in chronic hemodialysis patients,[3] and hemodialysis therapy improves some indices of HRV.[4]

Heart rate dynamics is a non-stationary, complex but a non-random process. Stationarity means that the statistical properties of the signal remain the same throughout the period of recording. Stationarity and periodicity are two fundamental assumptions of Fourier transform, a most frequently used linear HRV analysis method. However, both assumptions are not typical characteristics of heart rate dynamics. In addition, linear analysis method could not reveal the long-range organization and complexity embedded in heart rate dynamics.[5] The field of non-linear dynamics addresses the analysis of complex processes, and measures have been developed to describe the underlying structure of non-stationary, non-periodic but deterministic series of data. Detrended fluctuation analysis (DFA) is a scaling analysis method to represent the correlation properties of a signal [6]. The advantages of DFA over many other methods are that it permits the detection of long-range correlation embedded in seemingly non-stationary time series [7]. Studies have shown that DFA may provide more powerful information on the risk for fatal cardiovascular events [8,9].

We hypothesize that DFA is an important prognosis predictor in patients with ESRD receiving dialysis therapy. Since hemodialysis might have dramatic effects on heart beat dynamics both during and between therapies, we select patients with ESRD who received peritoneal dialysis (PD). Other well-known prognostic predictors are also measured for comparison.

## Materials and Methods

### Population

Between July 2007 and March 2009, 134 Taiwanese who had received PD with a conventional glucose-based lactate-buffered solutions (UltraBag; Baxter Healthcare SA, Singapore) for >6 months at National Taiwan University Hospital were consecutively enrolled. Patients with hepatic disease, cardiac myopathy, pericardial disease, or significant valvular heart disease (≥moderate), chronic obstructive pulmonary disease, chronic atrial fibrillation (AF), clinical signs of acute infection, prior renal transplant were excluded. As for the procedure of PD, peritoneal membrane transport characteristics were based on the result of the most recent peritoneal equilibration test, using the 4-hour dialysate-to-plasma creatinine concentration ratio (D/P_Cr_). PD adequacy was measured by peritoneal Kt/V. Residual renal function was measured with a 24-hour urine collection to calculate the renal Kt/V. A 24-hour ECG monitor (ZymedDigiTrak Plus 24 Hour Holter Monitor Recorder and Digitrak XT Holter Recorder 24 Hour, Philips, Amsterdam, Netherlands) and a standard transthoracic echocardiography (iE33 xMATRIX Echocardiography System, Philips, Amsterdam, Netherlands) were performed in each patient. All echocardiographic measurements were performed by the same cardiologist. Etiology of mortality was documented according to medical record. Written informed consent was obtained from every participant, and the study was approved by the institutional review board of the National Taiwan University Hospital.

### RR Interval Recordings

The 24-hour electrocardiography data were reviewed by an experienced technician with commercialized software (Zymed 2010 Holter Software). The QRS complexes were automatically classified and manually verified as normal sinus rhythm, atrial or ventricular premature beats, or noise by comparison with adjacent QRS morphologic features. The cardiac RR intervals were deduced from adjacent normal sinus beats. Missing intervals were interpolated with the cubic spline method.

### Time- and Frequency-Domain Parameters

The mean heart rate, standard deviation of N-N intervals (SDNN), and root mean square of successive differences of N-N intervals (RMSSD) were used as time-domain measures of HRV. The power spectrum densities were estimated by Welch’s averaged periodogram method.[10]. Very-low-frequency power (VLF, 0.0033 to 0.04Hz), low-frequency power (LF, 0.04–0.15Hz), and high-frequency power (HF, 0.15–0.4 Hz) were calculated from the entire 24-hour segment.

### Detrended Fluctuation Analysis

DFA quantifies fractal-like correlation properties of the time series data.[6] The root mean square fluctuations of the integrated and detrended data were measured within the observation windows of various sizes and then plotted against the size of the window on a log—log scale. The scaling exponent represents the slope of this line. In this study, both the short-term (DFAα1, 4 to 11 beats) and long-term (DFAα2, >11 beats) scaling exponents were calculated. All the analyses were performed by using software developed in-house provided by Matlab 7.9 (Mathworks, Inc., Natrick, Ma, USA).

### Statistical analysis

Continuous variables were expressed as mean ± standard deviation (SD) and categorical variables were expressed as percentages. Continuous variables were compared between groups of patients by using the Student’s-t test while the categorical variables were by Chi-square tests. The frequency domain HRV parameters were logarithmically transformed because their distributions were skewed. Causes of death other than cardiac can be considered a competing event of cardiac death. Univariate and multivariate competing risk model (subdistribution hazard) were used to obtain the hazard ratios for cardiac mortality and total mortality. Hypothesis test showed that results were compatible with proportional hazard assumption (P = 0.9952).[11–13] Variables that are statistically significant in univariate analysis were included in multivariate analysis. Cumulative incidence curves using competing risk model were plotted to show the survival trend between patients with high and low DFAα1. A P < 0.05 was considered statistical significance. All analyses were performed with SPSS 20.0 (SPSS Inc. Chicago, IL) and SAS, version 9.4 (SAS Institute Inc., Cary, North Carolina, USA).

## Result

After a median follow-up period of around 34 months, 14 patients died (11.7%), with 8 patients classified as cadiac mortality (7 patients died of ventricular arrhythmia and one of cardiogenic shock). Among the remaining six deaths, five deaths were due to sepsis, and one of unknown cause.

The basic characters of the study subjects are shown in Table 1. Age (63.1±9.5 vs. 52.5±12.4, P = 0.003), prevalence of coronary artery disease (CAD) (50.0% vs. 17.6%, P = 0.011) were higher in mortality group while prevalence of hypertension (HTN) (66.7% vs. 89.8%, P = 0.042), plasma hemoglobin level (9.41±1.00 vs. 10.20±1.33, P = 0.032), and renal Kt/V (0.04±0.08 vs. 0.19±0.27, P = 0.046) were higher in survival group. A borderline longer PD duration was noted in the mortality group (68.6 (7.1–102.1) vs. 29.6 (3.7–267.9) months, P = 0.056).

**Table 1:** Basic characteristics of the study subjects in mortality and survival groups.

		Mortality (N = 14)	Survival (N = 120)	P
**Risk factors**	Age	63.1±9.5	52.5±12.4	0.003
	Female, %	35.7	47.5	0.573
	PD duration, months	68.6 (7.1–102.1)	29.6 (3.7–267.9)	0.056
	BMI, kg/m^2^	23.5±3.8	23.3±3.5	0.818
	DM, %	28.6	20.0	0.490
	HTN, %	66.7	89.8	0.042
	Dyslipidemia, %	14.3	37.3	0.137
**Cardiovascular diseases**	CAD, %	50.0	17.6	0.011
	PAD, %	14.3	1.7	0.055
	Stroke, %	14.3	4.2	0.159
**Medications**	EPO, %	97.5	100	1.000
	ACEI. %	48.7	50.0	1.000
	Beta-blocker, %	60.5	42.9	0.255
	CCB, %	67.2	64.3	1.000
**Echocardiography**	LVEF, %	63.1±16.3	65.9±11.5	0.548
	LV mass, g	188.2±35.0	179.8±48.8	0.537
**Blood markers**	Log-CRP, mg/dL	-0.58±1.55	-1.06±1.55	0.276
	Hemoglobulin, g/dL	9.41±1.00	10.20±1.33	0.032
	Ca x P, mg^2^/dL^2^	54.34±9.94	51.63±14.55	0.499
	Albumin, g/dL	3.90±0.35	4.03±0.38	0.212
	Kt/V	2.02±0.28	2.07±0.31	0.564
	rKt/V	0.04±0.08	0.19±0.27	0.046
	nPCR, g/KgBW/d	0.92±0.19	0.96±0.20	0.419

ACEI, angiotensin-converting-enzyme inhibitor; BMI, body mass index; CAD, coronary artery disease; CCB, calcium channel blocker; CRP, C-reactive protein; DM, diabetes mellitus; EPO, erythropoietin; HTN, hypertension; LV, left ventricle; LVEF, left ventricular ejection fraction; nPCR, normalized protein catabolic rate; PAD, peripheral artery disease; PD, peritoneal dialysis; rKt/V, renal Kt/V.

The missing intervals interpolated with the cubic spline method accounted for 5% to 10% of all R-R intervals. There was no significant difference in time domain parameters between both groups (Table 2). In frequency-domain parameters, log-VLF (5.54±1.16 vs. 6.27±1.08, P = 0.019) and log-LF (3.77±1.76 vs. 4.56±1.31, P = 0.041) were significantly lower in the mortality group. In the DFA parameters, DFAα1 were significantly lower in the mortality group (0.89±0.20 vs. 1.18±0.29, P < 0.001).

**Table 2:** Linear and nonlinear heart rate variability parameters of the study subjects in mortality and survival groups.

		Mortality (N = 14)	Survival (N = 120)	P
**Time domain**	Mean NN	790.15±163.96	769.17±134.67	0.591
	SDNN	42.94±21.87	44.04±21.66	0.858
	RMSSD	21.68±20.16	15.09±12.18	0.251
**Frequency domain**	Log-VLF	5.54±1.16	6.27±1.08	0.019
	Log-LF	3.77±1.76	4.56±1.31	0.041
	Log-HF	3.72±1.80	3.74±1.21	0.972
**DFA**	α1	0.89±0.20	1.18±0.29	<0.001
	α2	1.20±0.19	1.21±0.14	0.931

DFA, detrended fluctuation analysis; HF, high frequency; LF, low frequency; NN, normal beat to normal beat; RMSSD, root mean square of successive differences of N-N intervals; SDNN, standard deviation of N-N intervals; VLF, very low frequency.

In Table 3, we divided patients into three groups with equal number to see trend for event for each HRV parameters and DFA. Significant trend was noted in LF/HF for total mortality (P for trend = 0.015), DFAα1 for cardiac mortality (P for trend = 0.010), and DFAα1 for total mortality (P for trend = 0.017). Patients with higher DFAα1 were associated with lower cardiac and total mortality. We searched cutoff value for DFAα1 using ROC curve analysis, and divided patients into two groups based on whether DFAα1 was higher than 0.95 or not since the AUC of DFAα1 (<0.95) to predict total mortality was 0.761 (95% C. I. = 0.617–0.905). Hazard ratios (HRs) using univariate subdistribution hazard model were shown in Table 4. DFAα1 ≥ 0.95 was significantly associated with both decreased cardiac mortality (HR: 0.042, 95% confidence interval (CI) = 0.005–0.333, P = 0.003) and total mortality (HR: 0.111, 95% CI = 0.036–0.348, P = 0.0002). For cardiac mortality, patients with CVD were also associated with increased risk (HR: 2.953, 95% CI = 1.849–4.715, p < 0.001). Higher rKT/V was associated with a trend toward lower risk, but the HR did not reach statistical significance (HR: 0,014, 95% CI = 0.000–2.157, P = 0.096). For total mortality, increased age (HR: 1.086, 95% CI = 1.039–1.136, P = 0.0003) and patients with CVD (HR: 2.299, 95% CI = 1.371–3.856, P = 0.002) were associated with increased risk. Patients with HTN (HR: 0.258, 95% CI = 0.082–0.818, P = 0.021), Hb ≥ 10,0 mg/dL (HR: 0.294, 95% CI = 0.093–0.927, P = 0.037), and patients with higher rKT/V (HR: 0,016, 95% CI = 0.000–0.400, P = 0.016) were associated with lower risk.

**Table 3:** Cox’s regression model by using HRV parameters as predictors for cardiac mortality and total mortality.

		Cardiac mortality					Total mortality				
		T2 vs. T1	p-value	T3 vs. T1	p-value	P for trend	T2 vs. T1	p-value	T3 vs. T1	p-value	P for trend
**Time domain**	SDNN	2.42(0.47,12.43)	0.29	0.48(0.04,5.27)	0.55	0.5293	1.67(0.49,5.68)	0.41	0.72(0.16,3.21)	0.67	0.6616
	RMSSD	0.00(0.00,0.00)	<.0001	0.90(0.23,3.47)	0.88	0.9164	0.48(0.09,2.55)	0.39	1.79(0.55,5.85)	0.34	0.3098
**Frequency domain**	Log-VLF	0.00(0.00,0.00)	<.0001	0.29(0.06,1.39)	0.12	0.1469	0.19(0.04,0.84)	0.029	0.27(0.08,0.99)	0.049	0.0514
	Log-LF	0.00(0.00,0.00)	<.0001	0.28(0.06,1.36)	0.12	0.1431	0.10(0.01,0.79)	0.029	0.36(0.11,1.13)	0.08	0.099
	Log_HF	0.47(0.09,2.49)	0.37	0.43(0.08,2.25)	0.32	0.3162	0.62(0.17,2.17)	0.45	0.54(0.16,1.84)	0.32	0.3284
	LF/HF	0.00(0.00,0.00)	<.0001	0.12(0.02,0.96)	0.046	0.0699	0.00(0.00,0.00)	<.0001	0.14(0.03,0.58)	0.0071	0.0154
**DFA**	α1	0.13(0.02,0.99)	0.049	0.00(0.00,0.00)	<.0001	0.0102	0.22(0.07,0.76)	0.0166	0.00(0.00,0.00)	<.0001	0.0002
	α2	0.34(0.04,3.27)	0.35	1.30(0.30,5.58)	0.73	0.7211	0.28(0.06,1.38)	0.12	0.68(0.22,2.10)	0.51	0.5128

DFA, detrended fluctuation analysis; HF, high frequency; LF, low frequency; NN, normal beat to normal beat; RMSSD, root mean square of successive differences of N-N intervals; SDNN, standard deviation of N-N intervals; T1, the first tertile; T2, the second tertile; T3, the third tertile; VLF, very low frequency.

**Table 4:** Univariate subdistribution hazard model by using clinical factors and DFAα1 as predictor for cardiac mortality and total mortality.

Variable	Cardiac mortality (n = 8)	p-value	Total mortality (n = 14)	p-value
**Age, years**	1.039(0.993,1.087)	0.102	1.086(1.039,1.136)	0.0003
**Gender, male**	1.263(0.321,4.971)	0.738	0.710(0.241,2.092)	0.535
**PD duration ≥ 30m**	1.550(0.375,6.399)	0.545	1.698(0.575,5.017)	0.338
**HTN**	0.837(0.103,6.785)	0.868	0.258(0.082,0.818)	0.021
**DM**	2.550(0.612,10.628)	0.199	1.801(0.559,5.796)	0.324
**CVD**	2.953(1.849,4.715)	<.0001	2.299(1.371,3.856)	0.002
**LVEF ≥ 50%**	0.342(0.084,1.397)	0.135	0.759(0.210,2.741)	0.674
**Hb≥ 10.0mg/dL**	0.784(0.203,3.035)	0.725	0.294(0.093,0.927)	0.037
**Albumin ≥ 4.0mg/dL**	0.688(0.176,2.700)	0.592	0.693(0.246,1.951)	0.487
**rKt/V**	0.014(0.000,2.157)	0.096	0.007(0.000,0.400)	0.016
**DFAα1 ≥ 0.95**	0.042(0.005,0.333)	0.003	0.111(0.036,0.348)	0.0002
**DFAα1**	0.05 (0.02, 0.19)	<0.0001	0.05 (0.01 0.19)	<0.0001

CRP, C-reactive protein; CVD, cardiovascular disease; DFA, detrended fluctuation analysis; DM, diabetes mellitus; Hb, hemoglobin; HTN, hypertension; LVEF, left ventricular ejection fraction; PD, peritoneal dialysis; rKt/V, renal Kt/V

**Fig 1:**
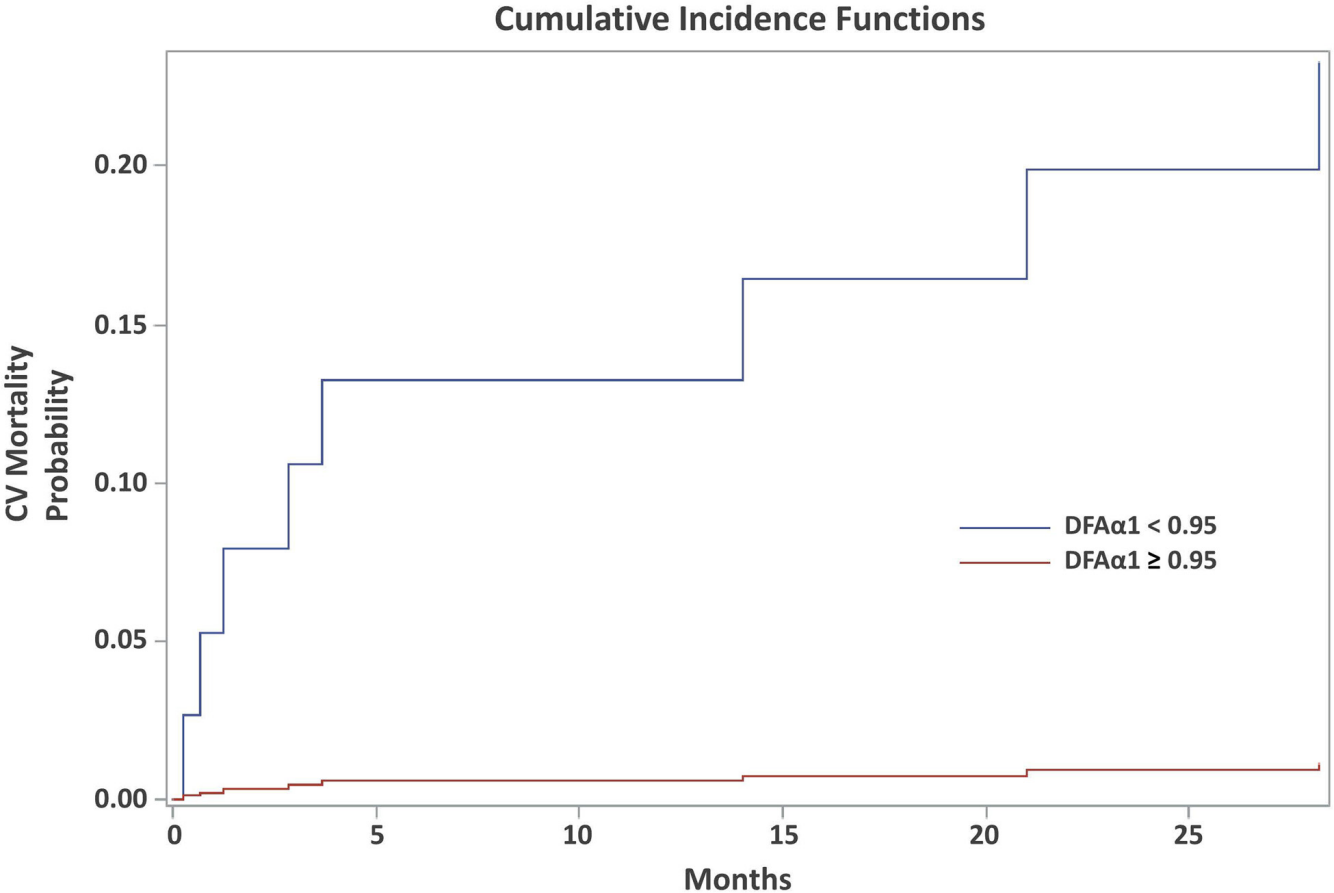
Cumulative incidence curve for cardiac mortality according to the contribution of DFAα1 using competing risk model. The survival significant decreased if the DFAα1 was below 0.95.

**Fig 2:**
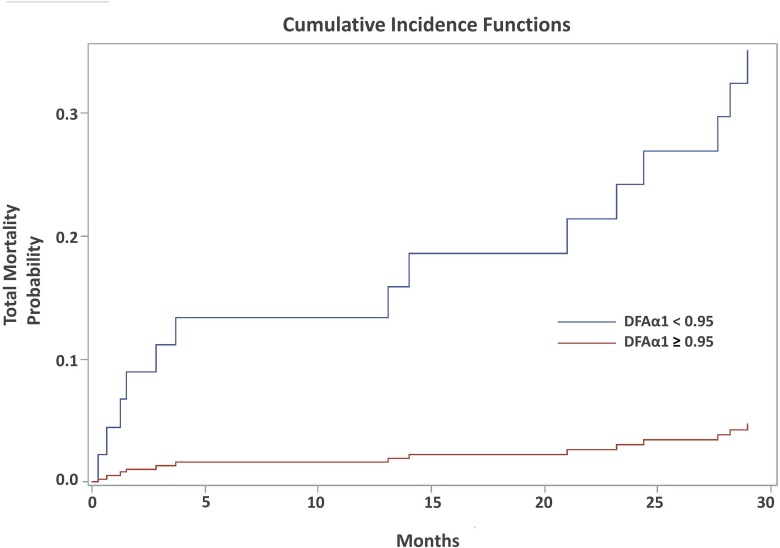
Cumulative incidence curve for total mortality according to the contribution of DFAα1 using competing risk model. The survival significant decreased if the DFAα1 was below 0.95.

In the multivariate subdistribution hazard model (Table 5), increased age (HR: 1.149, 95% C.I. = 1.069–1.236, P = 0.0002) and patients with CVD (HR: 4.245, 95% CI = 1.030–9.293, P = 00003) were associated with increased total mortality. Patients with HTN (HR: 0.210, 95% CI = 0.048–0.914, P = 0.038), higher rKT/V (HR: 0.000, 95% CI = 0.000–0.094, P = 0.015), and DFAα1 ≥ 0.95 (HR: 0,109, 95% CI = 0.033–0.362, P = 0.0003) were associated with decreased total mortality. DFAα1 ≥ 0.95 was also a significant predictor of lower risk for cardiac mortality (HR: 0.062, 95% CI = 0.007–9, = = 0.571, P = 0.014). In Figs 1 and 2, cumulative incidence of competing risk analysis for total and cardiac mortality according to the contribution of DFAα1 was shown. Total and cardiac mortality significant increased if the DFAα1 was below 0.95.

**Table 5:** Multivariate subdistribution hazard model by using clinical factors and DFAα1 as predictor for cardiac mortality and total mortality.

Variable	Cardiac mortality (n = 8)	p-value	Total mortality (n = 14)	p-value
**Age, years**			1.149(1.069,1.236)	0.0002
**HTN**			0.210(0.048,0.914)	0.038
**CVD**	1.939(1.127,3.333)	0.017	4.245(1.939,9.293)	0.0003
**Hb≥ 10.0mg/dL**			0.646(0.125,3.330)	0.602
**rKT/V**			0.000(0.000,0.094)	0.015
**DFAα1 ≥ 0.95**	0.062(0.007,0.571)	0.014	0.109(0.033,0.362)	0.0003

CVD, cardiovascular disease; DFA, detrended fluctuation analysis; Hb, hemoglobin; HTN, hypertension; rKt/V, renal Kt/V.

## Discussion

We examined the predicting value of various HRV parameters in patients with ESRD receiving PD, and demonstrated that lower DFAα1 is a strong predictor of both cardiac and total mortality. This is the first study to elucidate the dysregulation of autonomic system in patients with ESRD receiving PD by using DFA, and indicates that DFA could provide useful information for risk stratification in patients with ESRD receiving PD.

Increasing evidence has shown that HRV based on DFA might be more precise in predicting fatal arrhythmic events than that based on traditional methods in a variety of patient groups. For example, study has demonstrated that in post-myocardial infarction survivors with depressed left ventricular function, reduced DFAα1 was the most powerful predictor for all-cause mortality [9]. In general population with age over 65 years old, a reduced DFAα1 predicted the occurrence of sudden cardiac death [14]. Prior study had also shown that before onset of paroxysmal atrial fibrillation in patients without structural heart disease, significant changes in DFA values was demonstrated, whereas none of the time and frequency domain measures showed significant changes [15]. The reason that there was no significant association between DFAα1with cardiac mortality while the former being viewed as a continuous variable could imply the existence of a threshold value for DFAα1, below which the mortality increases rapidly.

Sympathetic over-activation may play an important role in the increased mortality in the above patient groups [16], and could be detected by DFA [17]. Patients with chronic kidney disease are also in a sympathetic overactive status [2]. In animal model, minor injury to the kidney induced by phenol injection caused central activation of the sympathetic nervous system.[18] In patients undergoing long-term maintenance hemodialysis, the sympathetic nerve discharge was higher than that in normal subjects, as shown by direct recording of the efferent sympathetic nerve discharge to the vasculature of the leg muscles [19]. Sympathetic overactivity increases intracellular cyclic AMP (cAMP), raises the rate of action potential generation in the sinoatrial (SA) node, and alters the beat-to-beat variability, as is reflected in changes in HRV. It can also alter the fractal heart rate dynamics by unbalancing the countervailing neuroautonomic inputs. One study has demonstrated that the fractal organization of human HR dynamics is determined by a delicate interplay between sympathetic and vagal outflow, with the breakdown of fractal HR behavior toward more random dynamics occurring during coactivation of sympathetic and vagal outflow [20]. The features of non-invasiveness and sensitivity made DFA an useful tool for prognostication of patient with ESRD receiving PD.

CV disease and infection disease are the two most common causes of death in patient with ESRD under dialysis, which consisted with the finding in our cohort. It is well established that uremia resulted in immune dysfunction, and prior study had proposed that atherosclerotic CV disease and infection could both be the result of immune dysfunction.[21] Interestingly, lower DFAα1predicted not only cardiac mortality, but also total mortality, which consisted of cardiac mortality and non-cardiac mortality, mostly contributed to sepsis. Whether patients with sympathetic overactivity are more vulnerable to infection disease, or lower DFAα1 indicates more pronounce immune dysfunction is unknown. In a way, DFA may provide a window to detect patients more susceptible to infection, and further study to address this issue is required.

There are two limitations in our study. First, we selected patient with ESRD receiving PD, which limited the generalization of the result to patients with chronic kidney disease not receiving PD because fluctuation of hemodynamics would be different in these patients. Second, we recruited only 134 patients having 8 cardiac mortality. Results might potentially be underpowered due to small sample size. In case a competing risk might hinder the observation of cardiac mortality, we used competing risk model. The relations between increased DFAα1 and cardiac or total mortality were consistently significant. As for clinical implication, use of DFA for prognostication of patient with ESRD receiving PD must be careful since DFA value is susceptible to other factors such as age and other comorbidity including AF. Besides, whether therapy to restore sympatho-vagal balance per se would provide clinical benefit or not remains an issue, which must be solved by clinical trials.

## Conclusion

Cardiac autonomic dysfunction evaluated by nonlinear HRV provided prognostic information in ESRD patients receiving PD. Increased DFAα1 is an independent predictor for lower cardiac and total mortality. Whether early intervention is needed in these high risk patients needs further confirmation.

## References

[1] Herzog CA, Mangrum JM, Passman R (2008) Sudden cardiac death and dialysis patients. Semin Dial 21: 300–307. 10.1111/j.1525-139X.2008.00455.x 18627568

[2] Vonend O, Rump LC, Ritz E (2008) Sympathetic overactivity—the Cinderella of cardiovascular risk factors in dialysis patients. Semin Dial 21: 326–330. 10.1111/j.1525-139X.2008.00456.x 18627567

[3] Oikawa K, Ishihara R, Maeda T, Yamaguchi K, Koike A, Kawaguchi H, et al (2009) Prognostic value of heart rate variability in patients with renal failure on hemodialysis. Int J Cardiol 131: 370–377. 10.1016/j.ijcard.2007.10.033 18199499

[4] Mylonopoulou M, Tentolouris N, Antonopoulos S, Mikros S, Katsaros K, Melidonis A, et al (2010) Heart rate variability in advanced chronic kidney disease with or without diabetes: midterm effects of the initiation of chronic haemodialysis therapy. Nephrol Dial Transplant 25: 3749–3754. 10.1093/ndt/gfq226 20466659

[5] Seely AJ, Macklem PT (2004) Complex systems and the technology of variability analysis. Crit Care 8: R367–384. 15566580 10.1186/cc2948 PMC1065053

[6] Peng CK, Havlin S, Stanley HE, Goldberger AL (1995) Quantification of scaling exponents and crossover phenomena in nonstationary heartbeat time series. Chaos 5: 82–87. 11538314 10.1063/1.166141

[7] Hu K, Ivanov PC, Chen Z, Carpena P, Stanley HE (2001) Effect of trends on detrended fluctuation analysis. Phys Rev E Stat Nonlin Soft Matter Phys 64: 011114 11461232 10.1103/PhysRevE.64.011114

[8] Ho KK, Moody GB, Peng CK, Mietus JE, Larson MG, Levy D, et al (1997) Predicting survival in heart failure case and control subjects by use of fully automated methods for deriving nonlinear and conventional indices of heart rate dynamics. Circulation 96: 842–848. 9264491 10.1161/01.cir.96.3.842

[9] Huikuri HV, Makikallio TH, Peng CK, Goldberger AL, Hintze U, Moller M (2000) Fractal correlation properties of R-R interval dynamics and mortality in patients with depressed left ventricular function after an acute myocardial infarction. Circulation 101: 47–53. 10618303 10.1161/01.cir.101.1.47

[10] Welch PD (1967) The Use of Fast Fourier Transform for the Estimation of Power Spectra: A Method Based on Time Averaging Over Short, Modified Periodograms. IEEE Transactions on Audio Electroacoustics AU-15: 70–73.

[11] Fine JP GR (1999) A proportional hazards model for the subdistribution of a competing risk. J Am Stat Assoc 94.

[12] Lim HJ, Zhang X, Dyck R, Osgood N (2010) Methods of competing risks analysis of end-stage renal disease and mortality among people with diabetes. BMC Med Res Methodol 10: 97 10.1186/1471-2288-10-97 20964855 PMC2988010

[13] Noordzij M, Leffondre K, van Stralen KJ, Zoccali C, Dekker FW, Jager KJ (2013) When do we need competing risks methods for survival analysis in nephrology? Nephrol Dial Transplant 28: 2670–2677. 10.1093/ndt/gft355 23975843

[14] Makikallio TH, Huikuri HV, Makikallio A, Sourander LB, Mitrani RD, Castellanos A, et al (2001) Prediction of sudden cardiac death by fractal analysis of heart rate variability in elderly subjects. J Am Coll Cardiol 37: 1395–1402. 11300452 10.1016/s0735-1097(01)01171-8

[15] Vikman S, Makikallio TH, Yli-Mayry S, Pikkujamsa S, Koivisto AM, Reinikainen P, et al (1999) Altered complexity and correlation properties of R-R interval dynamics before the spontaneous onset of paroxysmal atrial fibrillation. Circulation 100: 2079–2084. 10562264 10.1161/01.cir.100.20.2079

[16] Woo MA, Stevenson WG, Moser DK, Middlekauff HR (1994) Complex heart rate variability and serum norepinephrine levels in patients with advanced heart failure. J Am Coll Cardiol 23: 565–569. 8113535 10.1016/0735-1097(94)90737-4

[17] Tulppo MP, Makikallio TH, Seppanen T, Airaksinen JK, Huikuri HV (1998) Heart rate dynamics during accentuated sympathovagal interaction. Am J Physiol 274: H810–816. 9530192 10.1152/ajpheart.1998.274.3.H810

[18] Ye S, Zhong H, Yanamadala S, Campese VM (2006) Oxidative stress mediates the stimulation of sympathetic nerve activity in the phenol renal injury model of hypertension. Hypertension 48: 309–315. 16785328 10.1161/01.HYP.0000231307.69761.2e

[19] Converse RL Jr, Jacobsen TN, Toto RD, Jost CM, Cosentino F, Fouad-Tarazi F, et al (1992) Sympathetic overactivity in patients with chronic renal failure. N Engl J Med 327: 1912–1918. 1454086 10.1056/NEJM199212313272704

[20] Tulppo MP, Kiviniemi AM, Hautala AJ, Kallio M, Seppanen T, Makikallio TH, et al (2005) Physiological background of the loss of fractal heart rate dynamics. Circulation 112: 314–319. 16009791 10.1161/CIRCULATIONAHA.104.523712

[21] Kato S, Chmielewski M, Honda H, Pecoits-Filho R, Matsuo S, Yuzawa Y, et al (2008) Aspects of immune dysfunction in end-stage renal disease. Clin J Am Soc Nephrol 3: 1526–1533. 10.2215/CJN.00950208 18701615 PMC4571158

